# Hypotonic Activation of the Myo-Inositol Transporter SLC5A3 in HEK293 Cells Probed by Cell Volumetry, Confocal and Super-Resolution Microscopy

**DOI:** 10.1371/journal.pone.0119990

**Published:** 2015-03-10

**Authors:** Joseph Andronic, Ryo Shirakashi, Simone U. Pickel, Katherine M. Westerling, Teresa Klein, Thorge Holm, Markus Sauer, Vladimir L. Sukhorukov

**Affiliations:** 1 Department of Biotechnology and Biophysics, University of Würzburg, Biozentrum, Am Hubland, Würzburg, Germany; 2 Institute of Industrial Science, The University of Tokyo, Tokyo, Japan; The Hong Kong Polytechnic University, HONG KONG

## Abstract

Swelling-activated pathways for *myo*-inositol, one of the most abundant organic osmolytes in mammalian cells, have not yet been identified. The present study explores the SLC5A3 protein as a possible transporter of *myo*-inositol in hyponically swollen HEK293 cells. To address this issue, we examined the relationship between the hypotonicity-induced changes in plasma membrane permeability to *myo*-inositol *P*_ino_ [m/s] and expression/localization of SLC5A3. *P*_ino_ values were determined by cell volumetry over a wide tonicity range (100–275 mOsm) in *myo*-inositol-substituted solutions. While being negligible under mild hypotonicity (200–275 mOsm), *P*_ino_ grew rapidly at osmolalities below 200 mOsm to reach a maximum of ∼3 nm/s at 100–125 mOsm, as indicated by fast cell swelling due to *myo*-inositol influx. The increase in *P*_ino_ resulted most likely from the hypotonicity-mediated incorporation of cytosolic SLC5A3 into the plasma membrane, as revealed by confocal fluorescence microscopy of cells expressing EGFP-tagged SLC5A3 and super-resolution imaging of immunostained SLC5A3 by *direct* stochastic optical reconstruction microscopy (*d*STORM). *d*STORM in hypotonic cells revealed a surface density of membrane-associated SLC5A3 proteins of 200–2000 localizations/μm^2^. Assuming SLC5A3 to be the major path for *myo*-inositol, a turnover rate of 80–800 *myo*-inositol molecules per second for a single transporter protein was estimated from combined volumetric and *d*STORM data. Hypotonic stress also caused a significant upregulation of SLC5A3 gene expression as detected by semiquantitative RT-PCR and Western blot analysis. In summary, our data provide first evidence for swelling-mediated activation of SLC5A3 thus suggesting a functional role of this transporter in hypotonic volume regulation of mammalian cells.

## Introduction

Most animal cells are able to regulate their volume in anisotonic media [1,2]. Efficient volume regulatory mechanisms are essential for cell survival because they protect cells against excessive osmotic shrinkage or swelling [3]. In general, cell volume restoration is achieved by activation of volume-sensitive membrane pathways, which enable the cells to accumulate or to release osmotically active substances. In hypertonic media, shrunken cells undergo regulatory volume increase (RVI) via the uptake of extracellular electrolytes (e.g. NaCl) and osmotic water influx, whereas hypotonically swollen cells release KCl and shrink via regulatory volume decrease (RVD). A variety of channels and transporters are involved in the uptake or release of electrolytes (K^+^, Na^+^, Cl^-^, etc.) during cell volume regulation [4–8].

In addition to inorganic ions, a wide range of small organic osmolytes (SOOs), such as myo-inositol, sorbitol, taurine, etc., are important for maintaining the osmotic balance of cells [9–11]. A key role of these molecules in cell volume regulation is supported by the fact that various cell types possess considerable pools of SOOs [12]. Moreover, many mammalian cell types respond to hypertonicity by replacing cytosolic electrolytes with SOOs, thus avoiding detrimental effects of high ionic strength on structure and function of cytosolic proteins [9,13]. During hypertonic conditions, myo-inositol, as one of the most abundant SOOs, is synthesized by cells or accumulated from extracellular medium by the sodium-dependent cotransporter SMIT [1,5,14]. SMIT belongs to the solute carrier (SLC) superfamily and it is referred hereafter as SLC5A3, according to the established nomenclature [15].

Long-term exposure of cells to hypertonic solutions upregulates the transcription of SLC5A3 gene, which in turn leads to an increased SLC5A3-mediated uptake of *myo*-inositol [16,17]. There is a body of evidence in the literature indicating that the hypertonicity-induced expression of a battery of genes, including SLC5A3, crucial for the adaptation of mammalian cells to hypertonic stress is regulated by the tonicity enhancer-binding protein (TonEBP) or NFAT5 [18–21]. When expressed in *Xenopus* oocytes, SLC5A3 transports, besides myo-inositol, a wide range of substrates including glucose, galactose and others monomeric sugars and carbohydrates [22]. Compared to the mechanisms operating under chronic hypertonic conditions, the transport pathways involved in the release of *myo*-inositol and other SOOs from hypotonically swollen cells are much less understood [5]. Particularly, the potential impact of acute hypotonic stress on the expression and localization of SLC5A3 protein has not yet been investigated.

In previous studies on several mammalian cell types [23,24], we have demonstrated two distinct swelling-activated mechanisms differing in their osmotic thresholds and molecular selectivity. Particularly we have shown that moderate cell swelling under mild hypotonic conditions (≥200 mOsm) activates electrolyte pathways allowing the cells to accomplish RVD by releasing cytosolic ions. Neither myo-inositol nor other tested monomeric carbohydrates are able to permeate through the electrolyte pathways. But upon excessive cell swelling in 100-mOsm media, insertion of a yet-unknown pathway into the cell membrane from cytosolic vesicles renders it highly permeable to monomeric carbohydrates, including myo-inositol, sorbitol, glucose, galactose, etc. [23–25].

In view of its broad substrate specificity and tonicity-modulated expression mentioned above [16,17,22], the myo-inositol transporter SLC5A3 appears to be a promising candidate for a swelling-activated pathway for small carbohydrates. To test this hypothesis in the present study we analyzed the relationship between the amount of membrane-associated SLC5A3 protein and the hypotonically activated plasma membrane permeability to myo-inositol. To this end, we evaluated the membrane permeability coefficients for myo-inositol *P*_ino_ over a wide tonicity range (100–300 mOsm), by analyzing the volumetric response of HEK293 cells to hypotonic myo-inositol-substituted solutions. In parallel experiments, we applied confocal fluorescence microscopy to study cells expressing EGFP-tagged SLC5A3 and super-resolution imaging by *direct* stochastic optical reconstruction microscopy (*d*STORM) [26,27] to immunostained cells to examine the hypotonically induced changes in the intracellular and plasma membrane localization of myo-inositol transporters. In addition, we analyzed the impact of hypotonic stress on SLC5A3 gene expression using Western blot and semiquantitative RT-PCR.

## Material and Methods

### Cell culture

HEK293 cells were grown in DMEM with high glucose (4.5 g/L). The complete growth medium (CGM) also contained 10% fetal calf serum, 2 mM glutamine, 100 U/mL penicillin, 100 μg/mL streptomycin and 1% non-essential amino acids (all purchased from PAA Laboratories, Linz, Austria) in a humidified atmosphere (5% CO_2_) at 37°C. The cells were kept in exponential growth by replating three times a week.

### Hypotonic perfusion solutions and cell volumetry

*myo*-Inositol, sucrose and inorganic salts of the highest purified grade were purchased from Sigma (Munich, Germany). For cell volumetry, perfusion solutions of varying osmolalities (100, 125, …, 275, 300 mOsm) were used containing either myo-inositol or sucrose as the major osmolyte. In addition to the organic solute (∼85–285 mM), all perfusion solutions contained 0.9 mM magnesium acetate, 0.1 mM calcium acetate, and 12 mOsm K_2_HPO_4_/KHPO_4_, pH 7.4. The total osmolality of inorganic electrolytes was ∼15 mOsm. The solution osmolality was determined with a cryoscopic osmometer (Gonotec, Berlin, Deutschland).

Cell volume changes were monitored by video microscopy using a flow chamber designed for rapid medium exchange [23,25]. The chamber, fabricated of PDMS, was mounted on a microscope slide and sealed with a poly-d-lysine coated glass coverslip. Before measurements, cells were detached by shake-off without trypsin treatment. An aliquot of cell suspension in isotonic CGM (∼300 mOsm) at a density of ∼10^5^ cells/mL was injected into the chamber and the cells were allowed to settle and to adhere to the chamber floor. The chamber was placed on the stage of a microscope (Leica Leitz DMRM, Hamburg, Germany) and the cells were viewed with a 20x objective in transmitted light.

Cell volume changes were induced by perfusing the chamber with an inositol- or sucrose-substituted solution of varying osmolality (100–300 mOsm). The perfusion rate was set to 20 μL/s, using a syringe pump (KD Scientific, Holliston, USA). The cells were photographed every 10 s, starting 30 s before and up to 60 min after medium exchange with a CCD camera (uEYE, IDS GmbH, Obersulm, Deutschland) attached to the microscope. The response of cells to a strongly-hypotonic sucrose-substituted solution is illustrated by a time-lapse video in the Supporting material (S1 Video). The volume *V* of an individual cell was determined from its cross-section area assuming spherical geometry, using the software ImageJ (National Institutes of Health, Bethesda, Maryland). The cell volume was normalized to the original isotonic volume *V*_0_ as: *v* = *V*/*V*_0_. The mean *v* values (± SE, N = 20–40 cells) were calculated from a sequence of up to ∼360 microphotographs and plotted as functions of time. For each experimental condition, measurements were performed on 2–6 separate cell passages.

### Derivation of the membrane permeability coefficients for myo-inositol *P*_ino_

In addition to experiments involving a single perfusion, a separate set of measurements was conducted in which the same cell sample was perfused twice. The first perfusion with hypotonic sucrose solution (100–275 mOsm) gave rise, independent of the osmolality, to a rapid initial cell swelling followed by RVD. The occurrence of RVD implies that the plasma membrane remains impermeable to the disaccharide sucrose over the entire hypotonicity range. At different time intervals after the first perfusion (e.g. 5, 20 … or 40 min) the sucrose solution was replaced by an inositol-substituted medium of the same tonicity.

The *equiosmotic* replacement of sucrose by inositol at tonicities below 200 mOsm not only abolished RVD but also induced a considerable secondary cell swelling. Unlike the initial hypotonic swelling caused by an osmotic shift (e.g. 300 → 100 mOsm), the secondary swelling occurred under isosmotic conditions, i.e. *no* osmotic pressure gradient existed across the cell membrane. In our experiments, the isosmotic cell swelling implies an influx of the major extracellular solute myo-inositol into cells through swelling-activated pathways. In contrast, the isosmotic cell shrinkage during RVD involves the release of intracellular electrolytes.

As outlined in the Supporting Material (S1 Text), the isosmotic cell volume changes during RVD and secondary swelling can be used for the evaluation of membrane permeability coefficients, respectively, for electrolytes *P*_el_ and myo-inositol *P*_ino_. From the slope of cell shrinkage during RVD (d*v*/dt_RVD_) in a sucrose-substituted solution, we first calculated the *P*_el_ value, for each osmotic condition, by applying Eq. 1.

$$P_{el}=-\frac{R_{0}}{3}\frac{\left(C_{el}^{o}+C_{suc}^{o}\right)}{C_{suc}^{o}}{\left(\frac{dv}{dt}\right)}_{RVD}$$ (1)

where *R*_0_ = 7.9 μm is the mean radius of HEK293 cells; the extracellular concentration of electrolytes and sucrose were, respectively, $C_{el}^{o}\approx 15\;mOsm$ and $C_{suc}^{o}\approx 85-285\;mOsm$, according to the composition of perfusion solutions (see above).

We further determined the rate of secondary swelling d*v*/dt_ino_ induced by myo-inositol and applied Eq. 2 to calculate *P*_ino_:

$$P_{ino}=\frac{R_{0}}{3}\frac{\left(C_{el}^{o}+C_{ino}^{o}\right)}{C_{ino}^{o}}{\left(\frac{dv}{dt}\right)}_{ino}+P_{el}$$ (2)

where $C_{el}^{o}\approx 15\;mOsm$ and $C_{ino}^{o}\approx 85-285\;mOsm$. Equations 1 and 2 are derived in the Supporting Material section (S1 Text) using the two-parameter formalism [28,29].

The calculated *P*_ino_ values were plotted against the osmolality and fitted to a logistic sigmoid function of the form:

$$P_{ino}=P_{\min}+\frac{P_{\max}-P_{\min}}{1+{\left(C/C_{50}\right)}^{w}}$$ (3)

where *P*_min_, *P*_max_, and *w* are the parameters of the sigmoid. *C* is osmolality and *C*_50_ is the osmolality at which the myo-inositol permeability was of half-activated.

### RNA-Extraction

RNA extraction from hypotonically treated cells was performed using a peqGOLD TriFast kit (PEQLAB Biotechnologie GMBH, Erlangen, Germany). The adherently growing HEK293 cells were seeded in 6-well plates (2*10^5^ cells/well) and grown to 80–90% confluence. To induce hypotonic stress, the isotonic culture medium was replaced with diluted CGM (100 mOsm), in which the cells were incubated for 10, 20 or 30 min at room temperature. For RNA extraction, the hypotonic cell samples were lysed with 1 mL TriFast according to the manufacturer’s recommendations. The RNA pellets were dissolved in 50 μL of nuclease-free water (Sigma). The concentration of RNA was determined with a NanoPhotometer Pearl (Implen, Munich, Germany). After that, 1 u/μL of RNase inhibitor (RiboLock, Fermentas) was added to the samples and RNA was immediately used for cDNA synthesis. Isotonic controls were treated similarly, except for hypotonic treatment.

### cDNA Synthesis

A 1-μg aliquot of freshly prepared RNA was reverse transcribed (RT) into cDNA using RevertAid (Fermentas), following the manufacturer’s instructions. The reaction mixtures contained 0.5 mM dNTPs, 5X RT Buffer (Fermentas), 1 u/μL RiboLock Ribonuclease inhibitor (Fermentas) and 25 ng/μL random hexamers (Promega). Prior to cDNA synthesis, RNA samples, random hexamers and dNTP’s were incubated at 60°C for 5 min. The reaction was carried out at 42°C for 60 min in a C1000 Thermal Cycler (BioRad, Munich, Germany). After that, the samples were heated to 70°C for 10 min to denature the enzyme, and then cooled to 4°C.

### Semiquantitative RT-PCR

The mRNA levels of the genes encoding SLC5A3 and SLC6A6 were semi-quantified by the RT-PCR (reverse transcription PCR) method with respect to the mRNA level of transcripts of β-actin. Freshly prepared samples of cDNA from hypotonically treated cells were used for amplification of the genes SLC5A3 and actin using, respectively, the following primer sequences:

- SLC5A3 5’-GGATCC(BamHI)ATGAGAGCTGTACTGGACACAGCAGAC-3’ and 5’- GGATCC(BamHI)GCTAAGGAGAAATAAACAAACATGAAAATTC-3’
- ß- Actin 5’- GAATTC(EcoRI)GAAGCATTTGCGGTGGACG-3’ and 5’-GAATTC(EcoRI)ATGGATGATGATATCGCCGCGCTC-3’.

Two μL of cDNA products were typically amplified with 1 unit of Phusion DNA polymerase (Finnzyme) in the 5x HF buffer provided by the manufacturer and in the presence of 0.25 μM specific primers (purchased from biomers, Ulm, Germany) and 0.25 mM dNTPs. The optimum temperature for annealing of SLC5A3 primers was determined using a temperature gradient program. Reactions were carried out in a C1000 Thermal Cycler (BioRad, Munich, Deutschland). A first cycle of 30 s at 98°C was followed by 10 s at 98°C, 20 s at 48°C (SLC5A3) or 60°C (actin) and 50 s at 72°C for 26 cycles. A final extension step for 5 min at 72°C was followed by cooling to 4°C.

The amplified fragments were analyzed using ethidium bromide stained 1% agarose gels in sodium borate buffer [30]. A DNA molecular weight marker (FastRuler Middle Range DNA Ladder, Fermentas) was run on every gel to confirm the expected molecular weight of the PCR products. Images of the gels were acquired using a Gel iX-System (Intas, Göttingen, Deutschland) and quantification of the actin bands intensities were determined by ImageJ software (US National Institutes of Health). mRNA expression of SLC5A3 was normalized to β-actin. The correctness of the amplified SLC5A3 PCR product was verified by sequence analysis (GATC, Konstanz, Germany).

### Cloning

For construction of SLC5A3-EGFP, the SLC5A3 DNA band (30 minutes after hypotonic stress) from cDNA amplification was extracted from the agarose gel and blunt-end cloned into the cloning vector pJET (Fermentas) using the CloneJET PCR Cloning Kit (Fermentas). Afterwards the insert was cloned into the vector pEGFPN1 (Clonetech) in frame with the GFP coding sequence using the BAMHI restriction sites (restriction sites at the insert were delivered by the amplification primers). The correctness of SLC5A3-EGFP construct was verified by sequencing (GATC, Konstanz, Deutschland).

### Immunoblotting

Membrane protein extraction from hypotonically treated and isotonic cell samples was carried out using Mem-PER Plus Membrane Protein Extraction Kit (Pierce, Perbio, Bonn, Germany). The cells were seeded out into T25 cell culture flasks and grown to ∼90% confluence. Hypotonic stress was induced by substitution of the isotonic CGM with diluted 100-mOsm CGM. The cells were exposed to hypotonicity for 10, 20, and 30 min at room temperature. Thereafter, membrane proteins were extracted from isotonic and hypotonic samples, according to the manufacturer’s recommendations. Protein concentrations were determined using the RotiQuant Universal assay (Roth, Karlsruhe, Germany). For each experimental condition, equal amounts of protein (∼10 μg) were subjected to SDS-PAGE on 10% polyacrylamide gels and blotted onto a nitrocellulose membrane (life technologies, Darmstadt, Germany). After the transfer, the membranes were stained with Ponceau-S solution (0.5% Ponceau-S in 1% acetic acid) and analyzed as described elsewhere [31]. The Ponceau-S stained membranes were scanned with a flatbed scanner (Canon LiDE 110), and the blot images were analyzed with the ImageJ software. The blots were then blocked with 5% bovine serum albumin in TBST (1% Tween-20 in TRIS buffered saline), for 1h at room temperature. Thereafter the blots were incubated with the primary antibody rabbit anti-SLC5A3 (ab110368; abcam, Cambridge, UK; 1:1000) over night at 4°C. After washing, the blots were incubated for 1 h at room temperature with a horseradish peroxidase conjugated anti-rabbit antibody (New England Biolabs; 1:2000). Bound antibodies were visualized with tetramethyl benzidine (TMB) solution for immunoblots (life technologies, Darmstadt, Germany). Western blots were scanned (Canon LiDE 110), and band densities were determined using the ImageJ software. The results were corrected for background and protein loading differences (determined by Ponceau-S staining).

### Confocal laser microscopy

HEK293 cells were transiently transfected with the SLC5A3-EGFP construct using FuGene HD (Promega) according to the manufacturer’s instructions, in μ-slide flow chambers (IBIDI, Munich, Germany). Microscopic analysis was performed ∼30 hours after transfection.

Confocal fluorescence images were taken with an LSM 710 using a Plan-Apochromat 63x/1.40 oil immersion objective (Zeiss, Jena, Germany) and argon laser light excitation at 488 nm. Fluorescence intensity distribution was quantified using ImageJ software. The cross section through a hypotonic cell was selected to match that for isotonic conditions. Hypotonicity was applied to cells by replacing isotonic CGM with 100-mOsm myo-inositol-substituted medium. Confocal images were analyzed with the *plot profile* function of ImageJ software.

### *d*STORM (*direct* stochastic optical reconstruction microscopy)

To investigate the amount of native SLC5A3 protein present in the plasma membrane of HEK293 cells we used single-molecule based localization microscopy by *d*STORM [26,27,32]. The experimental setup for *d*STORM was described in detail previously [27,33]. Prior to the measurements, 5*10^4^ cells were seeded on poly-d-lysine coated eight-well Lab-Tek II chambered cover glasses (Nunc, Wiesbaden, Germany). Cells were detached by shake-off and rinsing with fresh CGM, without trypsin treatment. Cells were allowed to settle and to adhere for 1 h at 37°C, and then washed twice with PBS. Hypotonic shock was applied by substitution of isotonic PBS with 100-mOsm inositol- (or sucrose-) substituted medium. Control isotonic samples were incubated in PBS. Cell fixation without permeabilization was performed by treatment with 4%(v/v) formaldehyde (Sigma) for 30 min.

Cells were then washed with PBS, and incubated for 1h at room temperature with 5% (w/v) BSA (Sigma) in PBS to prevent nonspecific antibody binding. Afterwards, cells were incubated for 30 min in 5% (w/v) BSA in PBS comprising 1:500 mouse mAB anti-SLC5A3 clone 3A6 (SAB1402920, Sigma) at room temperature. Specificity of the primary antibody was verified by comparing the SLC5A3-EGFP-signal with the fluorescence signal derived from immunolabeling with Alexa Fluor 568 (data not shown). According to the SLC5A3 protein structure predicted in [34] the primary antibody recognizes an extracellular epitope. The cells were then washed thoroughly with PBS and incubated in Alexa Fluor 647-conjugated F(ab′)2 fragments of goat anti-mouse-IgG (A-21237, Invitrogen) 1:200 diluted in 5% (w/v) BSA in PBS for 20 min at room temperature. The negative control sample was incubated with the secondary antibody alone. Afterwards cells were thoroughly washed with PBS and post-fixated in 4% (v/v) formaldehyde in PBS for 10 min.

*d*STORM of Alexa Fluor 647 was performed in 100 mM β-mercaptoethylamine (MEA; Sigma) in PBS, pH 7.4–8.0 (with KOH), containing an oxygen scavenger system of 5–10 u/ml glucose oxidase (Sigma), ∼120 u/ml catalase (Sigma) and 4% w/v glucose [27]. To illuminate only a thin membrane layer highly inclined illumination mode was used. For excitation of Alexa Fluor 647 a laser emitting at 639 nm (Genesis MX STM 640; Coherent, USA) was used. For each *d*STORM image, 15.000 frames were recorded with an exposure time of typically 15 ms and at a frame rate of ∼65 Hz and constant irradiation intensity ranging between 1 and 5 kW cm^-2^.

For data processing and image reconstruction, the open access software for single-molecule–based localization microscopy rapidSTORM 3.2 [35,36] was used as previously described [27]. For analysis only fluorescent spots that contain more than 769 photons were used. Multi-fluorophore events were identified by point-spread function analysis [36] and discarded. For each cell, the density of Alexa Fluor 647 localizations was calculated as the number of localized fluorophores per cellular membrane cross-section area, determined from the corresponding transmitted light image.

## Results

### Swelling activated myo-inositol permeability

**Fig 1:**
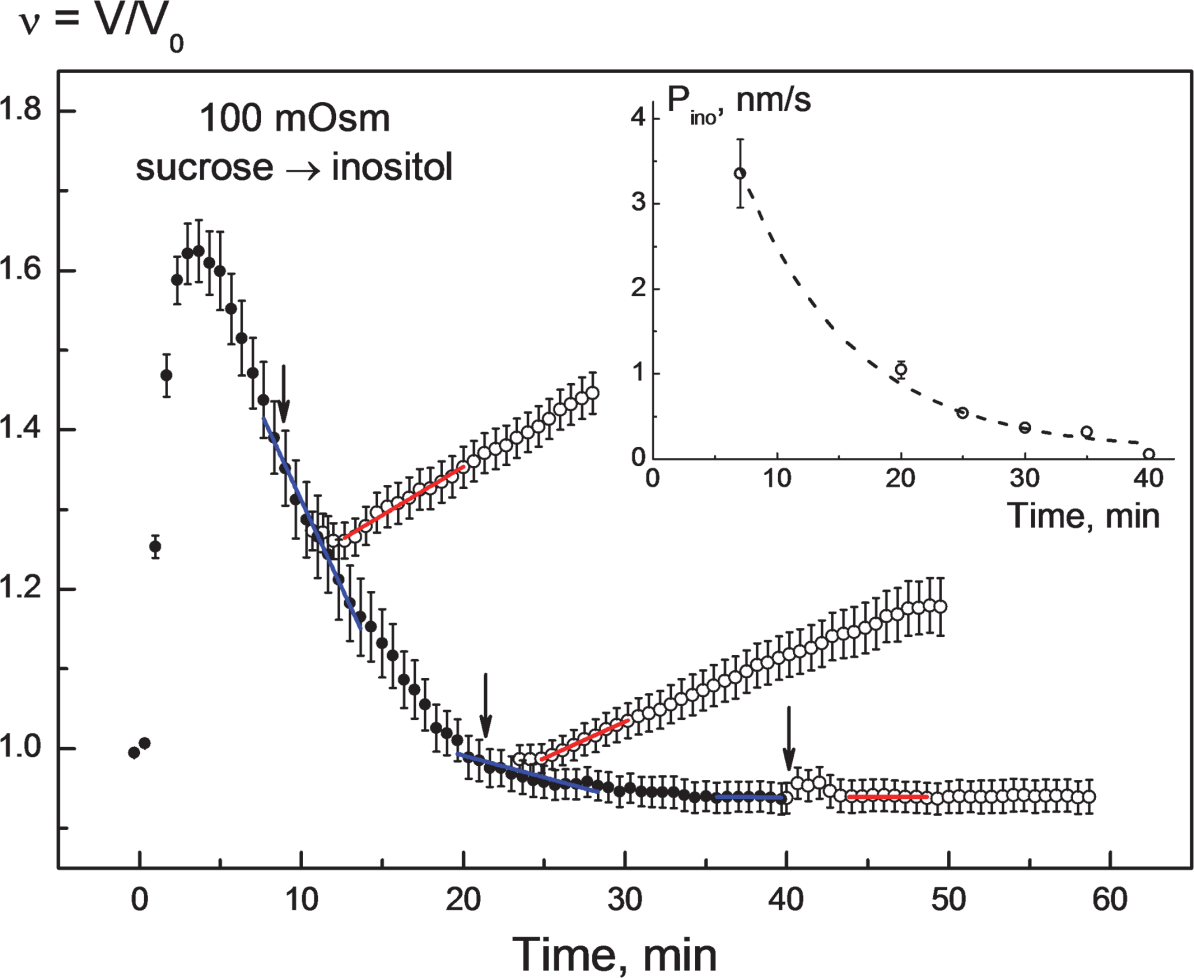
Changes in the normalized volume *v* = V/V_0_ of HEK293 cells in response to sequential application of sucrose and *myo*-inositol solutions of the same osmolality of 100 mOsm. The cells were bathed initially (*time* < ∼30 s) in isotonic growth medium (300 mOsm) and then exposed to a 100-mOsm sucrose solution. The strongly hypotonic sucrose solution (*filled symbols*) caused fast cell swelling to a transient maximum volume *v*_max_ of ∼1.6 within the first 2–3 min. After the initial swelling, the cells underwent RVD, i.e. they gradually shrank to reach the original isotonic volume (*v*_0_ ≈ 1) within ∼20 min in the presence of sucrose. The replacement of sucrose by an equiosmotic amount of *myo*-inositol (*arrows*) abolished RVD and caused secondary cell swelling (*empty symbols*). The rate of secondary swelling (Δ*v*/Δ*t*_ino_, *red fitted lines*) decreased with time during and after RVD (7–35 min). The addition of *myo*-inositol 40 min after hypotonic shock did not cause any significant cell swelling. Each data point represents the mean ± SE of 25–42 individual cells measured in 2–3 independent experiments. For each time point of *myo*-inositol addition, the rates of RVD Δ*v*/Δ*t*_RVD_ (*blue lines*) and the rates of secondary swelling Δ*v*/Δ*t*_ino_ (*red lines*) were determined to calculate the permeability coefficients for myo-inositol *P*_ino_ by applying Eqs. 1 and 2. The inset illustrates the decay of *P*_ino_ with time during RVD.

In agreement with previous studies on numerous mammalian cell lines [23,25], hypotonically swollen HEK293 cells are capable of RVD in strongly hypotonic sucrose-substituted solutions (Fig. 1). As seen in Fig. 1 (*filled symbols*), 100-mOsm sucrose solution caused the cells to swell rapidly within ∼2–3 min from their isotonic volume (*v*_0_ = 1) to a transient maximum *v*_max_≈1.6. After that the cells shrank gradually and recovered their original isotonic volume within ∼20 min after hypotonic shock. The occurrence of RVD clearly shows that, despite their permeability to cytosolic electrolytes, swelling-activated membrane pathways in HEK293 cells are impermeable to the disaccharide sucrose.

At different time intervals after application of 100-mOsm sucrose solution, i.e. during and after RVD, cells were perfused with a myo-inositol-substituted solution of the same osmolality (*arrows* in Fig. 1). Although no osmotic shift was applied, the equiosmotic replacement of sucrose by myo-inositol during RVD gave rise to a rapid secondary swelling of cells, as illustrated by the empty symbols in Fig. 1. The observed isosmotic swelling indicates that the myo-inositol influx rate into cells exceeds that of the RVD-related efflux of intracellular solutes. The fastest secondary swelling with a rate Δ*v*/Δ*t*_ino_ of ∼1.8 10^–4^ s^-1^ occurred if inositol was added shortly after the onset of RVD (*t* ≈ 7 min). Thereafter, Δ*v*/Δ*t*_ino_ progressively decreased the later inositol was applied, e.g. to ∼1.5 10^–4^ s^-1^ at 20 min. After ∼40 min, 100-mOsm myo-inositol did not cause any significant cell swelling. The Δ*v*/Δ*t*_ino_ values were determined from the slopes of the red fitted lines (Fig. 1). The corresponding RVD rates Δ*v*/Δ*t*_RVD_ (indicated by *blue lines*) were ∼8 10^–4^, 2 10^–4^ and 0 s^-1^, respectively, for the time intervals 7, 20, and 40 min after hypotonic shock.

By applying Eqs. 1 and 2 to the Δ*v*/Δ*t*_RVD_ and Δ*v*/Δ*t*_ino_ data derived from the volumetric curves shown in Fig. 1, we calculated *P*_ino_ values during RVD and plotted them against the total time of hypotonic exposure (*inset* in Fig. 1). For these calculations we used a mean radius of HEK293 cells *R*_0_ = 7.9 μm, $C_{el}^{i}=100\;mOsm$, and $C_{el}^{o}=15\;mOsm$, according to our experimental conditions. Shortly after the onset of RVD (*t* ≈ 7 min), *P*_ino_ was ∼3.3 nm/s. After that, *P*_ino_ decreased steadily with time and vanished at ∼40 min after hypotonic shock. These data clearly show that after a transient activation of *myo*-inositol pathways by hypotonic swelling, cells restore their original membrane impermeability to myo-inositol during RVD.

**Fig 2:**
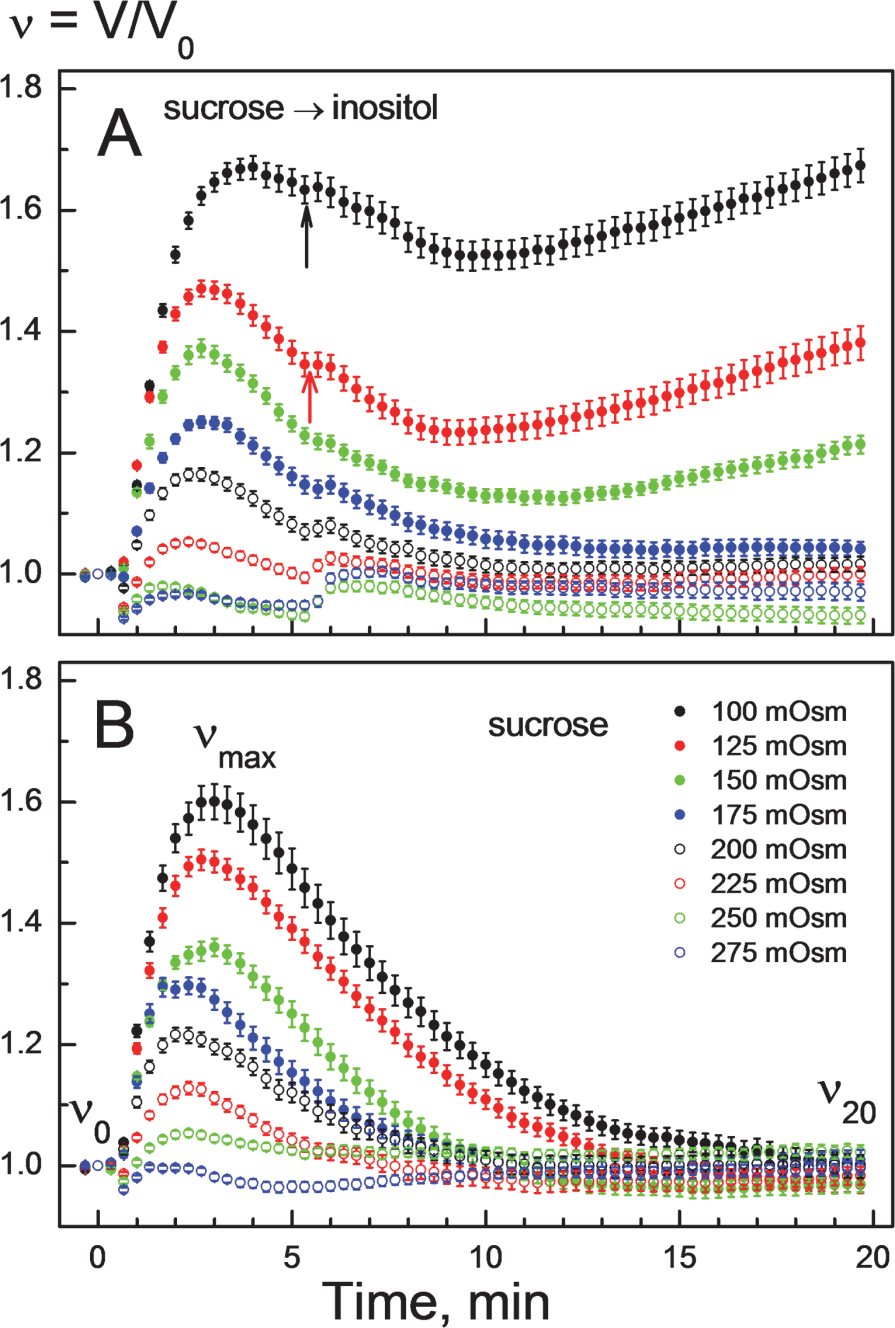
Volume changes of HEK293 cells in response to solutions of varying osmolality and composition. At time ∼30 s, the cells were first transferred from isotonic growth medium to a sucrose-substituted solution having osmolality of 100, 125, … 250 or 275 mOsm. Thereafter, the hypotonic sucrose solutions were replaced at time ∼5 min with myo-inositol solutions of the same osmolalities (***A***). In contrast, the cells were exposed for 20 min to sucrose solutions only (***B***). After the initial swelling in the presence of sucrose, the cells were capable of RVD over the entire hypotonicity range (*B*). A nearly complete RVD also occurred in slightly hypotonic solutions of myo-inositol (*A*, ∼175–275 mOsm). Application of more diluted myo-inositol solutions (100–150 mOsm; *t* ≈ 5–9min) considerably inhibited cell shrinkage via RVD. Thereafter (*t* ≈ 9–20 min) the cells exhibited sustained secondary swelling (***A***), which is indicative of myo-inositol uptake by cells. For each tonicity, the rates of RVD Δ*v*/Δ*t*_RVD_ and secondary swelling Δ*v*/Δ*t*_ino_ were used to calculate the permeability coefficients for electrolytes *P*_el_ and inositol by applying Eqs. 1 and 2, respectively.

To analyze the impact of osmolality on swelling-activated membrane permeability to myo-inositol, we conducted volumetric experiments by varying the tonicity of perfusion solutions over a wide range, i.e. from mildly hypotonic (e.g. 275 mOsm) to strongly hypotonic conditions (100 mOsm). In the experiments illustrated in Fig. 2A, the cells were first exposed to a hypotonic sucrose solution, which was replaced, shortly after the onset of RVD (*t* ≈ 5 min), by a myo-inositol solution of the same osmolality. For comparison, Fig. 2B shows the volumetric data of cells treated with hypotonic sucrose solutions only. Independent of osmolality, the disaccharide allowed RVD in HEK293 cells over the entire tonicity range studied (Fig. 2B).

Under mild hypotonic conditions of 200–275 mOsm, isosmotic replacement of sucrose by myo-inositol had little, if any, effect on the RVD of HEK293 cells (*open symbols* in Fig. 2A). But at osmolalities below 175 mOsm, myo-inositol not only abolished RVD but also induced secondary cell swelling (*filled symbols* in Fig. 2A). The cells achieved the fastest swelling rates (Δ*v*/Δ*t*_ino_) not immediately but with a delay of about 4-min after application of myo-inositol. After that (*t* > 9 min), cell volume increased linearly with time. Therefore, we derived the Δ*v*/Δ*t*_ino_ values for the time interval 9–14 min after hypotonic shock. At tonicities between 175 and 275 mOsm, cell swelling was negligible (Δ*v*/Δ*t*_ino_ ≈ 0), whereas at 150, 125 and 100 mOsm the Δ*v*/Δ*t*_ino_ values were 0.2 10^−4^, 1.8 10^−4^ and 1.9 10^−4^ s^−1^, respectively. The corresponding rates of RVD (Δ*v*/Δ*t*_RVD_), derived from the data shown in Fig. 2B for the time interval 10 ± 2 min, were ∼4 10^−4^, 6.8 10^−4^, 7.8 10^−4^ s^−1^ in 150-, 125- and 100-mOsm sucrose solutions, respectively.

**Fig 3:**
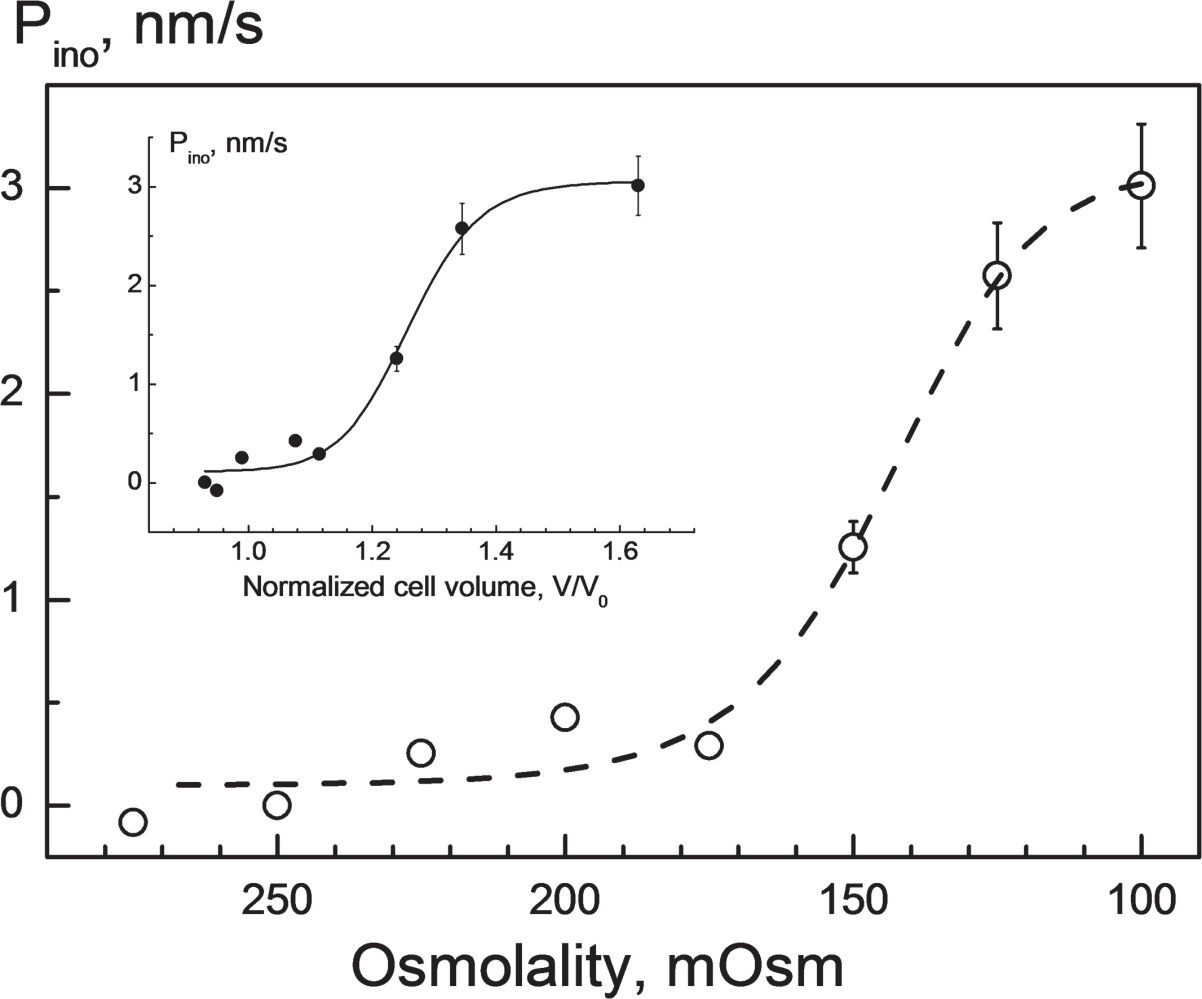
Impact of hypotonicity on the myo-inositol permeability *P*_ino_ in HEK293 cells. The *P*_ino_ values were calculated using Eq. 2 from the rates of secondary swelling, using the volumetric data shown in Fig. 2A. The fit of Eq. 3 to the data yielded a *C*_50_ value of 144 ± 10 mOsm, i.e. the tonicity at which the myo-inositol permeability was half-activated. In the inset, the same *P*_ino_ data are plotted as function of the cell volume at the time point of *myo*-inositol application. Curve fitting (Eq. 3) shows that *P*_ino_ was half-activated as cells swelled by about 26% (*v*_50_ = 1.26±0.02).

By substituting the Δ*v*/Δ*t*_RVD_ values into Eq. 1, we first calculated the permeability coefficients for electrolyte *P*_el_ [nm/s] during RVD at different osmolalities. For these calculations we used $C_{el}^{i}=(100\;-275)\;mOsm$ and $C_{el}^{o}=15\;mOsm$, according to our experimental conditions. After that, the permeability coefficients for myo-inositol *P*_ino_ [nm/s] were calculated by applying Eq. 2 and using the corresponding *P*_el_ values and Δ*v*/Δ*t*_ino_ data of myo-inositol-mediated swelling. The dependence of *P*_ino_ on osmolality is illustrated in Fig. 3. In contrast to the inset in Fig. 1, which illustrates the reduction of *P*_ino_ with time during RVD in 100-mOsm sucrose solution, Fig. 3 shows near-maximal *P*_ino_ values revealed shortly after the onset of RVD in cells exposed to a wide range of tonicities (Fig. 2).

As seen in Fig. 3 (*symbols*), *P*_ino_ was relatively low, i.e. between ∼0 and ∼0.3 nm/s, under mild hypotonic conditions of 175–275 mOsm. But decreasing osmolality below 175 mOsm resulted in a rapid increase of *P*_ino_, e.g. to ∼3 nm/s at 100 mOsm. Fitting the data to a sigmoidal function (Eq. 3) yields a *C*_50_ value of 144 ± 10 mOsm corresponding to the tonicity of half-maximal activation of *myo*-inositol permeability. The inset in Fig. 3 shows the same *P*_ino_ data plotted versus the cell volume *v* = V/V_0_ during *myo*-inositol application. Fitting with Eq. 3 gives a *v*_50_ value of ∼1.26, indicating that half-activation of *P*_ino_ was induced by an about 26% increase in volume of hypotonically swollen cells.

### Intracellular localization of SLC5A3-EGFP affected by hypotonicity

In the following experiments we address the solute carrier protein SLC5A3 as a possible candidate for a swelling-activated transporter of myo-inositol in cells exposed to severe hypotonic conditions. We have shown previously [23–25] that transport pathways for a range of small carbohydrate molecules including the polyols myo-inositol and sorbitol are inserted into the plasma membrane from cytosolic vesicles via swelling-mediated exocytosis in strongly hypotonic solutions (∼100 mOsm). This finding prompted us to analyze the impact of hypotonic shock on the intracellular localization of the myo-inositol transporter SLC5A3. To this end, we constructed an SLC5A3-EGFP fusion protein and overexpressed it in HEK293 cells by transient transfection. Thirty hours after plasmid transfection, we evaluated the expression and localization of SLC5A3-EGFP in cells by confocal laser scanning microscopy. Endogenous expression of SLC5A3 mRNA in HEK293 cells was proved in separate experiments using semiquantitative RT-PCR (*see* below).

**Fig 4:**
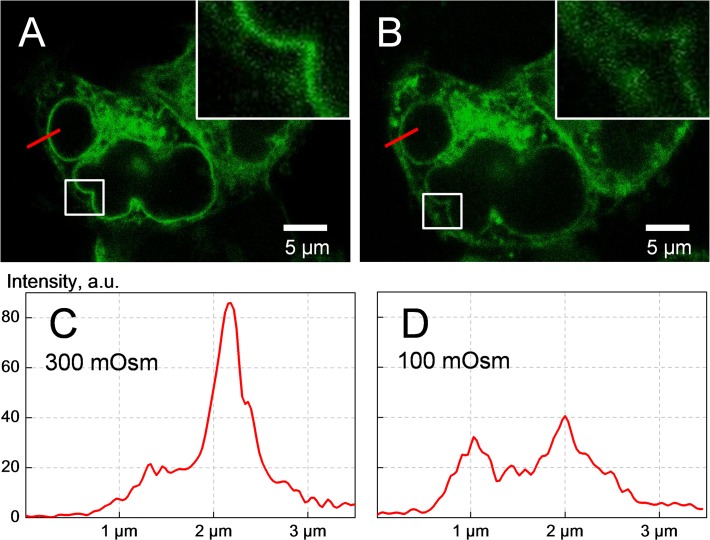
Confocal fluorescence imaging of HEK293 cells overexpressing the fusion protein SLC5A3-EGFP. The images *A* and *B* were taken, respectively, under isotonic conditions and 10 min after application of a strongly hypotonic 100-mOsm myo-inositol-substituted solution. Hypotonic cell swelling is clearly seen in (*B*). The insets and the intensity diagrams (*C* and *D*) illustrate the impact of hypotonic stress on the intracellular distribution of the fusion protein. Comparison of the diagrams *C* and *D* reveals a marked hypotonicity-mediated depletion of the protein in the perinuclear regions along with its increase in the peripheral cytoplasm. Together, these findings suggest that hypotonic swelling caused translocation of a large portion of SLC5A3-EGFP towards the plasma membrane.

As evident from the microphotographs shown in Fig. 4, the transfected cells express the fusion protein mainly in the cytoplasm, whereas the nuclei are practically devoid of fluorescence. Moreover, under isotonic conditions (Fig. 4A), the fluorescence is mainly localized in the endoplasmic reticulum and close to the nuclear envelope, which seems to be typical for overexpressed membrane proteins [37]. In contrast, the dim fluorescence of the peripheral cytoplasm suggests that only a small portion of the fusion protein resides in/near the plasma membrane in control isotonic cells. The subcellular protein distribution in isotonic cells, presented by the intensity diagram in Fig. 4C (along the radial red-colored lines indicated in Fig. 4A) clearly shows a major perinuclear peak with a magnitude of ∼85 a.u. at x ≈ 2.2 μm and a minor shoulder at x ≈ 1.2 μm corresponding to the peripheral cytoplasm/plasma membrane.

Besides the expected increase in cell size (compare Fig. 4B vs 4A), the exposure of cells to a strongly hypotonic myo-inositol solution (100 mOsm) causes marked changes in the subcellular localization of SLC5A3-EGFP. Comparison of the radial intensity distributions revealed that the perinuclear fluorescence (x≈2 μm in Fig. 4C and 4D) decreases by ∼50%, i.e. from the isotonic 85 a.u. to the hypotonic 40 a.u. At the same time, the plasma membrane peak increases from ∼20 to 30 a.u. The intensity diagrams clearly show that hypotonic shock leads to considerable displacement of SLC5A3-EGFP from the perinuclear area towards the plasma membrane. This effect is also evident from the magnified views of the marked box areas given in the insets of Fig. 4A and 4B.

### Hypotonicity-induced changes in SLC5A3 protein associated with the plasma membrane

**Fig 5:**
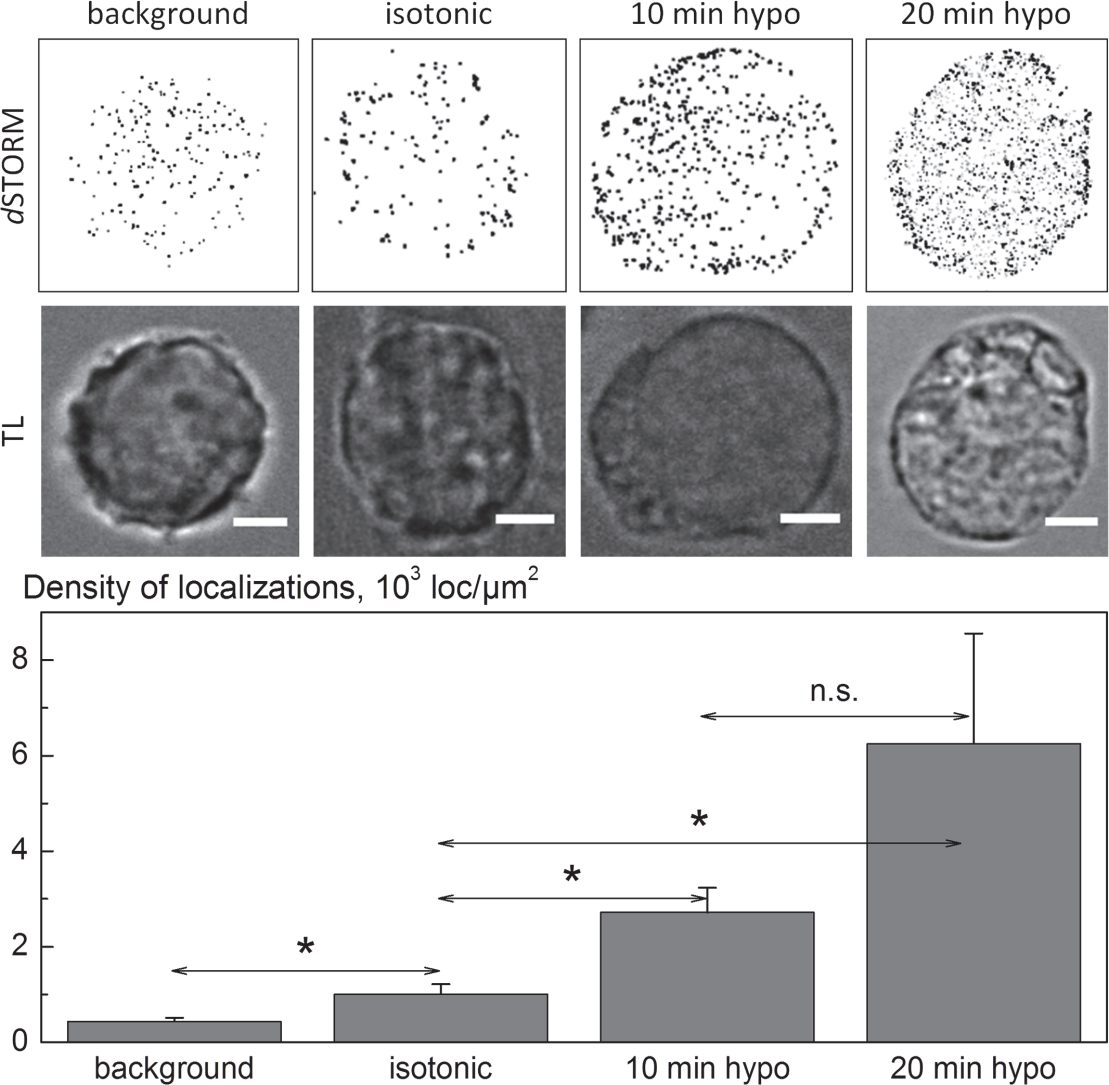
*d*STORM imaging of immunolabeled SLC5A3 protein in the plasma membrane of HEK293 cells under isotonic and hypotonic conditions. Images of the same cells in transmitted light (TL) are also shown. From the *d*STORM images (reconstructed from 15,000 single frames), the surface membrane density of SLC5A3 localizations [loc/μm^2^] were identified in individual cells. The bar graph shows the impact of hypotonic stress on the surface membrane density of SLC5A3 protein localizations. The data are means (±SD) from 8–16 individual cells for each osmotic condition and hypotonic stress duration. The differences in the mean values between the isotonic control and the two hypotonic samples were statistically significant (as denoted by *; *P* < 0.05), according to the Mann-Whitney test conducted using the Software Origin 9 (Microcal, Northampton, MA). The difference between the two hypotonic samples (10 vs 20 min) was not significant (n.s.).

The fluorescence images in Fig. 4 suggest, but do not definitely prove, that hypotonic swelling is accompanied by the insertion of SLC5A3-EGFP into the plasma membrane. Therefore, we further analyzed the impact of hypotonic stress on the amount of native SLC5A3 protein associated with the plasma membrane of HEK293 cells by super-resolution imaging. In the experiments illustrated by Fig. 5, we exposed the cells to a 100-mOsm inositol solution for 10 and 20 min. Cells treated with isotonic PBS served as control. To ensure selective labeling of membrane-associated SLC5A3 proteins, we used a primary antibody directed against an extracellular epitope of SLC5A3 protein. Prior to *d*STORM imaging, we applied a secondary antibody conjugated with the photoswitchable fluorescent dye Alexa Fluor 647.

As illustrated by the representative images in Fig. 5, hypotonic cells exhibit an increased amount of membrane-associated SLC53A molecules, as compared to isotonic control. The amounts of SLC53A localizations found in single cells, such as shown in in Fig. 5, were subject to some variation. Therefore, we compared the mean numbers of SLC53A localizations per unit membrane area (localization densities, [loc/μm^2^]) from 8–16 individual cells for each osmotic condition. As evident from the bar graph in Fig. 5, the mean density of SLC5A3 localizations in isotonic cells is comparable to the background level observed after staining with the secondary antibody only.

Application of a strongly hypotonic myo-inositol solution for 10 and 20 min, respectively, results in an ∼3 and 6-fold increase in localization density. The observed increase with time might have been associated with the continuous swelling of cells exposed to 100-mOsm myo-inositol (*see* Fig. 2B), which was used for the *d*STORM analysis illustrated in Fig. 5. The membrane-associated SLC5A3 in cells undergoing RVD in 100-mOsm sucrose showed completely different kinetics (Supporting information, S1 Fig.). Thus, comparison of the *d*STORM and volumetric data reveals that 5 min after hypotonic exposure transiently swollen cells (*v*_max_ ≈ 1.6, Fig. 1B) exhibit the highest density of SLC5A3 localizations (1.5 10^2^ loc/ μm^2^, S1 Fig.). But during RVD, e.g. 10 min after hypotonic shock, the level of membrane-associated SLC5A3 decreases to 0.4 10^2^ loc/μm^2^ (S1 Fig.) as the cells shrink to *v*_10_ ≈ 1.2 (see Fig. 1).

### Impact of hypotonicity on the expression of SLC5A3

**Fig 6:**
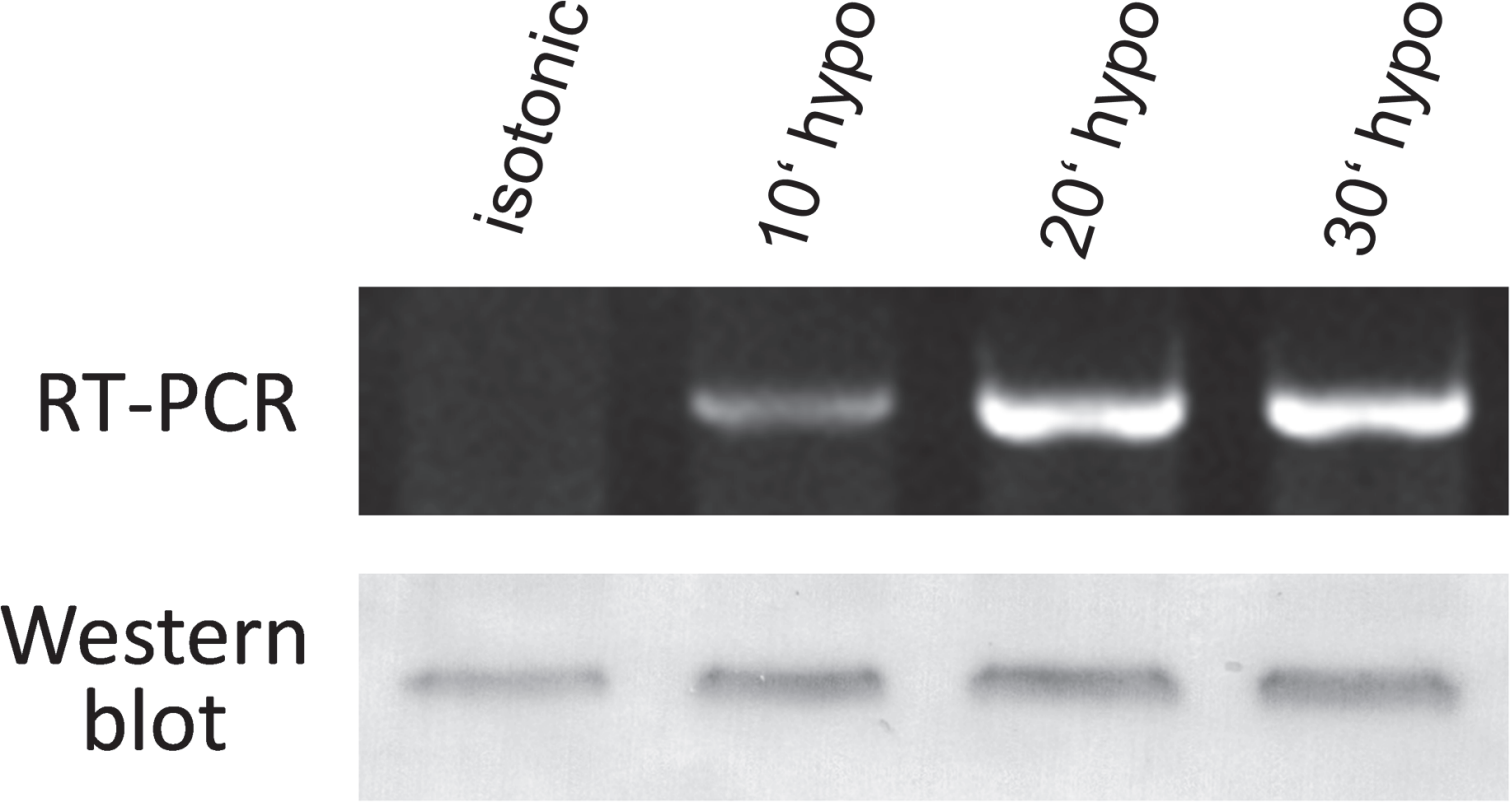
Hypotonic stress-induced upregulation of SLC5A3 at the mRNA and protein level in HEK293 cells revealed by semiquantitative RT-PCR and Western blot, respectively. Prior to RNA and protein extractions, the cells were incubated in 100-mOsm CGM for 10–30 min. Control cells were kept in isotonic CGM. The SLC5A3 mRNA level in isotonic sample was negligible, whereas hypotonicity induced substantial amounts of SLC5A3 mRNA. As with RT-PCR, Western blot analysis shows increased amounts of SLC5A3 protein (by up to ∼40%, *see* text) in hypotonic samples. For RT-PCR, β-actin was used as a template loading control (*see* Supporting information, S2 Fig., upper image). Prior to immunoblotting, reversible Ponceau-S protein staining has been used as a loading control (S2 Fig., lower image).

The results presented above clearly show that acute hypotonic stress induces massive changes in the cell membrane permeability to myo-inositol within few minutes (Figs. 1–3). These changes are very likely associated with a rapid incorporation of the myo-inositol transporter SLC5A3 into the plasma membrane from the preexisting cytosolic pool (Figs. 4 and 5). To prove whether SLC5A3 gene transcription and translation are also triggered by hypotonicity we analyzed the impact of hypotonic treatment on the expression of SLC5A3 gene. Prior to RNA and protein extractions, aliquots of HEK293 cells were exposed to hypotonic 100-mOsm CGM. At different time intervals of hypotonic treatment (10, 20 and 30 min), the total mRNA and membrane proteins were extracted and analyzed, respectively, by semiquantitative RT-PCR and Western blot. As seen in Fig. 6 (upper part), the expression of mRNA for SLC5A3 was negligible in isotonic control cells, whereas Western blot analysis revealed a detectable isotonic level of SLC5A3 protein.

Incubation of HEK293 cells in hypotonic solution for 10–30 min gave rise to a significant upregulation of the expression of SLC5A3 mRNA (Fig. 6, upper part). Within the same time interval, hypotonic stress also led to a moderate increase in the SLC5A3 protein level as illustrated by the representative Western blot in Fig. 6 (lower part). After hypotonic treatment for 10, 20 and 30 min, the normalized SLC5A3 protein band densities were increased with respect to isotonic control, respectively, by 43±3%, 39±14%, and 21±4% (means±ranges of two independent experiments). Together, the results in Fig. 6 demonstrate a significant hypotonic activation of the SLC5A3 gene expression in HEK293 cells at both transcriptional and translational levels.

## Discussion

The volumetric data presented in Figs. 1 and 2A clearly show that below a tonicity threshold of ∼200 mOsm myo-inositol causes marked secondary swelling of HEK293. In sharp contrast, the disaccharide sucrose allows the cells to accomplish RVD even under severe hypotonic conditions (Fig. 1 and 2B). The secondary swelling is a clear-cut indication of *myo*-inositol influx through swelling-activated pathways. This explanation is consistent with previous reports demonstrating that various small solutes, including monomeric carbohydrates, amino acids, organic and inorganic ions inhibit RVD via swelling-activated uptake of these molecules by cells [25,38,39].

As evident from Fig. 2A, a significant activation of myo-inositol permeability requires at least a 50% reduction of external osmolality from 300 to 150 mOsm. This finding corroborates the results of previous studies demonstrating that in various cell lines/types the transport of myo-inositol, sorbitol and other monomeric carbohydrates usually occurs when cells are exposed to strongly hypotonic solutions. For example, human neuroblastoma cells have been reported to release ^3^H-inositol at tonicities of 200 mOsm and below [40]. Similarly, the efflux rate of ^3^H-inositol from primary rat astrocytes has been found to increase rapidly with decreasing osmolality from 180 to 100 mOsm [41], which agrees very well with our *P*_ino_ data presented in Fig. 3. Consistent with the efflux studies, the influx of myo-inositol and sorbitol into Jurkat lymphocytes has been activated by cell swelling in strongly hypotonic 100-mOsm solution, but not at 200 mOsm [23,25]. Moreover, various monomeric sugars (glucose, deoxy-glucose, galactose) and small polyethylene glycols (PEG300–400) also permeate the plasma membrane of a wide range of cell types (e.g. Jurkat lymphocytes, human glioblastoma, human dendritic cells) exposed to strongly hypotonic solutions of ∼75–100 mOsm [24,42–44].

In the present study, the plasma membrane of HEK293 cells swollen by 60–70% (*v*_max_≈1.6–1.7; Figs. 1 and 2) in a 100-mOsm solution exhibits a permeability coefficient for myo-inositol *P*_ino_ of ∼3 nm/s (Fig. 3). Interestingly, this *P*_ino_ value derived here from the kinetics of cell swelling driven by myo-inositol influx agrees well with the permeability coefficient of ∼1.5 nm/s obtained for the efflux of H^3^-labeled sorbitol from IMCD cells swollen by ∼50% [45]. Moreover, transiently activated by cell swelling, the membrane permeability to both solutes decays rapidly during RVD, as illustrated for myo-inositol by the inset in Fig. 1 and for H^3^-sorbitol by Fig. 1 in [45].

Hypotonicity induced changes in the expression/localization of the myo-inositol transporter SLC5A3 revealed here by confocal fluorescence microscopy (Fig. 4), *d*STORM (Fig. 5) and mRNA determination (Fig. 6) strongly suggests this protein as a possible pathway underlying the swelling-activated membrane permeability to myo-inositol (Figs. 1–3). SLC5A3 protein, aka Na^+^ myo-inositol transporter SMIT, has been reported to be activated in response to hypertonic conditions [13,46,47]. In the present study we provide the first evidence that acute hypotonic stress induces massive changes in the expression and intracellular localization of SLC5A3. These changes include (i) the displacement of a large portion of the protein from the cytosolic compartments towards the plasma membrane (Fig. 4), (ii) the incorporation of SLC5A3 into the plasma membrane (Fig. 5), and (iii) upregulation of SLC5A3 gene expression (Fig. 6). An important finding is that both the swelling-activated membrane permeability and SLC5A3 protein displacement occurs on the same time scale, i.e. around 10 min, after hypotonic shock.

*d*STORM analysis of membrane-associated SLC5A3 reveals ∼2 10^3^ fluorophore localizations per μm^2^ in HEK293 cells exposed for 10 min to 100-mOsm myo-inositol (Fig. 5). Cells treated with 100-mOsm sucrose exhibit a much lower SLC5A3 density (∼2 10^2^ loc/∙μm^2^, S1 Fig.). The discrepancy is most likely due to the very different cell volume responses to strongly hypotonic myo-inositol and sucrose solutions, respectively, in which cells exhibit secondary swelling (Fig. 2A) and accomplish RVD, during which the myo-inositol permeability rapidly decays (Fig. 1).

Although fluorescence labeling of SLC5A3 with primary and secondary antibodies is neither quantitative nor stoichiometric, the observed localization densities can serve as a first useful estimate for the number of transporter molecules per unite membrane area (*n*_SLC_). Assuming that in our experiments the membrane-associated SLC5A3 provided the major route for the uptake of myo-inositol by hypotonically swollen cells, we can assess the myo-inositol transfer rate by a single SLC5A3 transporter. The transporter rate is denoted hereafter as *k*_ino_ [myo-inositol molecules s^−1^]. The following equation relates the molar myo-inositol flux *J* [mol s^−1^ m^−2^] through the membrane to myo-inositol permeability *P*_ino_ [m s^-1^], transporter rate *k*_ino_ and SLC5A3 surface density *n*_SLC_:

$$J_{ino}=P_{ino}\Delta c_{ino}=k_{ino}n_{slc}/N_{A}$$ (4)

where *N*_A_ stands for the Avogadro constant (6.02 10^23^ mol^-1^). Equation 4 transforms into the following expression for *k*_ino_:

$$k_{ino}=P_{ino}\Delta c_{ino}N_{A}/n_{slc}$$ (5)

To calculate *k*_ino_, we used a *P*_ino_ value of 3 nm/s and a membrane surface density of SLC5A3, *n*_SLC_ = (0.2–2)×10^3^ proteins/μm^2^, both derived for HEK293 cells, respectively, from the volumetric and *d*STORM experiments (Figs. 3 and 5). The initial difference in inositol concentration across the membrane Δ*c*_ino_ was ∼85 mmol/L (see Materials and Method). Substitution of these experimental data into Eq. 5 yields an estimate for the transporter rate *k*_ino_ of ≈ 80–800 myo-inositol molecules/s.

Until now SLC5 proteins with known functions have commonly been analyzed by electrophysiological techniques because these proteins can operate as electrogenic cotransporters [48]. Particularly, the SLC5A3 protein expressed in *Xenopus* oocytes has been reported to cotransport myo-inositol with a *K*_m_ of ∼50 μM and Na^+^ with a *K*_m_ in the 10-mM range [15,48]. On the other hand, members of the SLC5 family are also known to behave as Na^+^ uniporters, solute and water channels [15]. In addition to the reported mechanisms, the results of our volumetric experiments conducted in Na^+^-free solutions (Figs. 1–3) suggest the facilitated diffusion (uniport) as the most likely mechanism for the SLC5A3-mediated influx of myo-inositol in the presence of large extracellular concentrations of this solute.

As pointed out elsewhere [49], the rate of solute translocation across the membrane is an important experimental criterion distinguishing transporters from channels. Whereas a single ion channel usually allows the passage of 10^6^–10^8^ ions/s, a membrane transporter operates at a much lower rate, typically within the range of 10^2^–10^3^ solute molecules/s (see [49], p. 274). Judging from the moderate transfer rate of 80–800 substrate molecules/s found here, SLC5A3 protein appears to function as a transporter rather than a channel.

Besides the effects on the membrane permeability and SLC5A3 localization discussed above, hypotonic shock induces a marked increase in the expression of SLC5A3 gene at both transcriptional and translational levels (Fig. 6). Interestingly, the increase in gene activity occurs on a relatively short time scale within 10–20 min after hypotonic shock (Fig. 6), i.e. within the same time range as the observed changes in membrane permeability (Figs. 1–3) and SLC5A3 protein localization density (Figs. 4–5). The short-term hypotonic upregulation of SLC5A3 gene expression observed here might be a transient phenomenon, since a long-term hypotonic treatment (e.g. for 3–18 h) leads to a strong downregulation of SLC5A3 mRNA in mammalian cells [19].

Taken together, our data provide several lines of evidence that the myo-inositol transporter SLC5A3 is activated by an acute hypotonic shock. In view of the large cytosolic concentrations of myo-inositol in mammalian cells (∼1 mM) [25], this carrier protein can play an important role in the RVD process in a variety of cell types and lines. During RVD, SLC5A3 can provide a route not only for the release of cytosolic myo-inositol, but also for a wide range of related monomeric carbohydrates, including glucose, galactose, etc., as demonstrated for this protein expressed in *Xenopus* oocytes [22].

**Fig 7:**
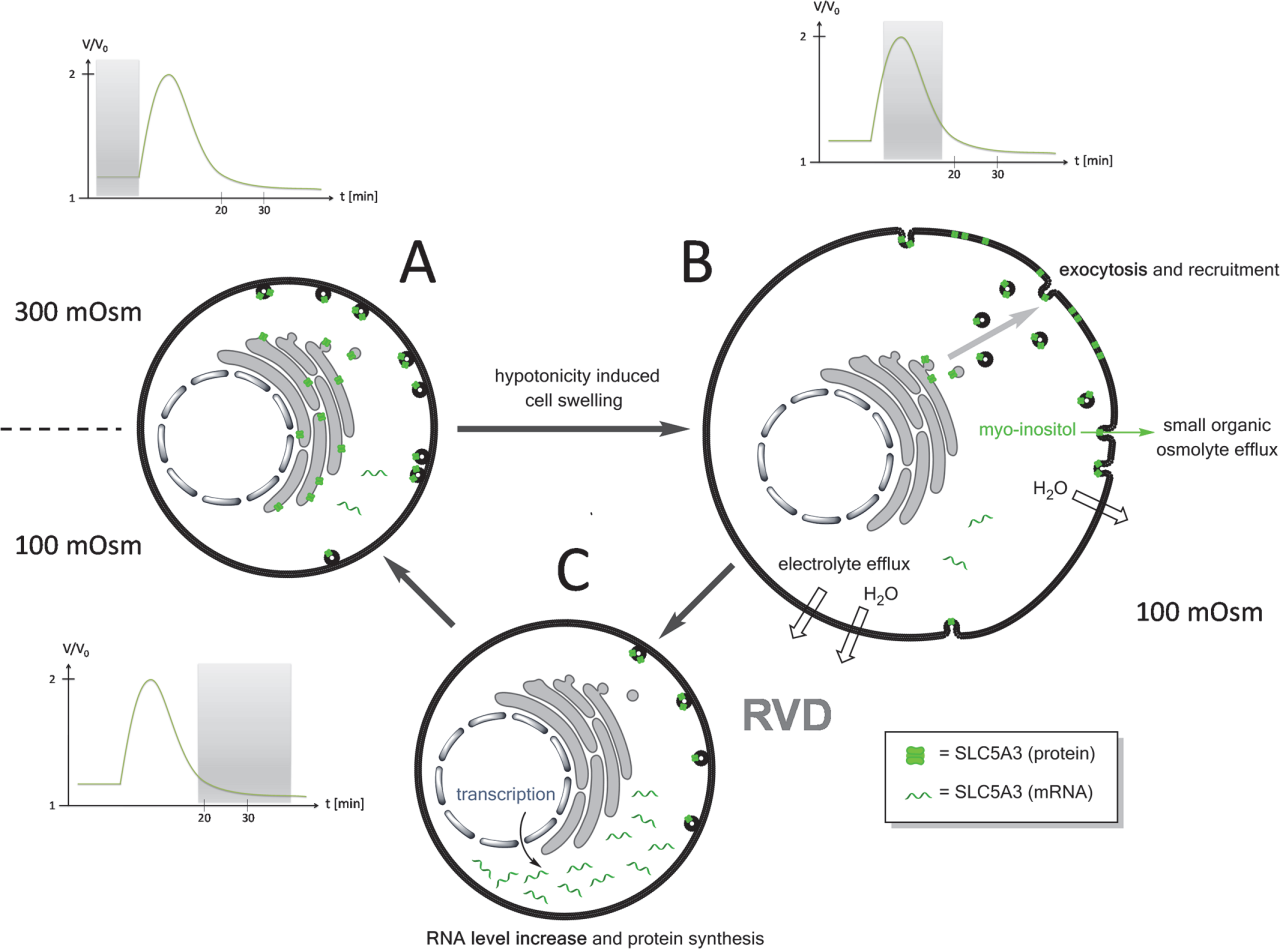
A putative mechanism of SLC5A3-mediated myo-inositol release during RVD. **A:** Under isotonic conditions the SLC5A3 protein is mainly localized in the cytosolic vesicles and its mRNA level is low. The plasma membrane permeability to SOOs and electrolytes is poor. **B:** Exposure of cells to a strongly hypotonic solution, e.g. 100-mOsm sucrose, causes rapid volume increase followed by cell shrinkage via RVD (see Fig. 1). The swelling-associated increase in the plasma membrane area is achieved (i) by unfolding of microvilli and (ii) by exocytotic fusion of cytosolic vesicles [23]. The fusion of vesicles carrying SLC5A3 protein leads to the incorporation of this transporter into the plasma membrane. The SLC5A3-mediated efflux of myo-inositol and related SOO contributes to cell shrinkage (RVD) and restoration of the original isotonic cell volume. **C:** During RVD, the cells recover the original membrane impermeability to myo-inositol (Fig. 1), presumably, via the endocytosis of excessive plasma membrane along with reinternalization and, possibly, lysosomal degradation of SLC5A3. The increased mRNA and protein expression of SLC5A3 (Fig. 6) suggest that *de novo* synthesis of SLC5A3 occurs to restore the depleted cytoplasmic pool of the transporter.

Based on the results presented here and previous studies [23,24] we propose the following model for the involvement of SLC5A3 in the RVD process (Fig. 7). The model includes several events triggered by the initial cell swelling in response to a tonicity decrease below a threshold of ∼150 mOsm (Fig. 3). In the first step, the membrane area increases via the swelling mediated exocytosis (fusion) of cytosolic vesicles [23]. The incorporation of SLC5A3 proteins from vesicles renders the plasma membrane permeable to myo-inositol. In the absence of extracellular SOOs, the cells undergo RVD by releasing myo-inositol and related compounds from the cytosol. In 100-mOsm sucrose medium, the cells accomplish RVD within 20 min after hypotonic shock (Fig. 1B). During RVD, the cells recover the original membrane impermeability to myo-inositol (Fig. 3), presumably, via the endocytosis of excessive plasma membrane along with re-internalization and, possibly, lysosomal degradation of SLC5A3.

Exocytosis and vesicular fusion appear to be common activation mechanisms among the SLC family members. Many physiologically important SLC transporters, including the glucose transporter GLUT4 (SLC2A4), as well as the neurotransmitter transporters SLC18A1, SLC18A2 and SLC18A3 are known to be regulated by exocytosis [50,51]. The betaine-GABA-transporter SLC6A12 (BGT-1), involved in the transport of the SOO betaine, has been shown to be relocated from cytosol to the plasma membrane upon hypertonic stimulation [52]. Other SLC-proteins operating as SOO transporters (e.g. SLC6A6, SLC6A12, etc.) can also be expected to be activated during cell volume regulation via similar mechanisms involving exocytosis.

In conclusion, several experimental approaches, including cell volumetry, EGFP tagging, immunocytochemistry, mRNA determination and Western blot analysis, confocal and super-resolution fluorescence microscopy used here provided new insights into the mechanisms of swelling-activated transport of the small organic osmolyte myo-inositol. Whereas the volumetric data yielded information on the osmotic thresholds for membrane permeability, the combination of confocal and subdiffraction-resolution microscopy were useful for monitoring the hypotonicity-induced changes in the intracellular distribution of the myo-inositol transporter SLC5A3. Although this study focuses on the hypotonically induced uptake of myo-inositol, similar regulatory mechanisms for the transport of structurally dissimilar organic solutes mediated by different SLC transport systems can also be operational in a wide range of mammalian cell types.

## Supporting Information

S1 Fig *d*STORM imaging of immunolabeled SLC5A3 protein in the plasma membrane of HEK293 cells under isotonic (PBS) and hypotonic conditions. In these experiments, the cells were treated with a strongly hypotonic 100-mOsm sucrose solution for 5 and 10 min. In contrast to the experiments with 100-mOsm myo-inositol presented in Fig. 5, the cells underwent RVD in the presence of the disaccharide sucrose, as evident from Fig. 1B. (For further detail see Legend to Fig. 5)
(TIF)

S2 Fig Loading controls for semiquantitative RT-PCR (β-actin) and Western blot (Ponceau-S staining) analyses. For further detail *see* text and Legend to Fig. 6.
(TIF)

S1 Video A time-lapse video clip illustrating the response of HE293 cells to a strongly-hypotonic sucrose-substituted medium. The cells were bathed initially in isotonic growth medium (300 mOsm) and then exposed (at zero time) to a 100-mOsm sucrose solution.
(AVI)

S1 Text Model of swelling-activated myo-inositol transport (DOCX)
