# Supplementary figures and images for: Hypotonic Activation of the Myo-Inositol Transporter SLC5A3 in HEK293 Cells Probed by Cell Volumetry, Confocal and Super-Resolution Microscopy
Source: PLoS One. 2015 Mar 10;10(3):e0119990. doi: 10.1371/journal.pone.0119990 (PMC4355067; doi:10.1371/journal.pone.0119990)

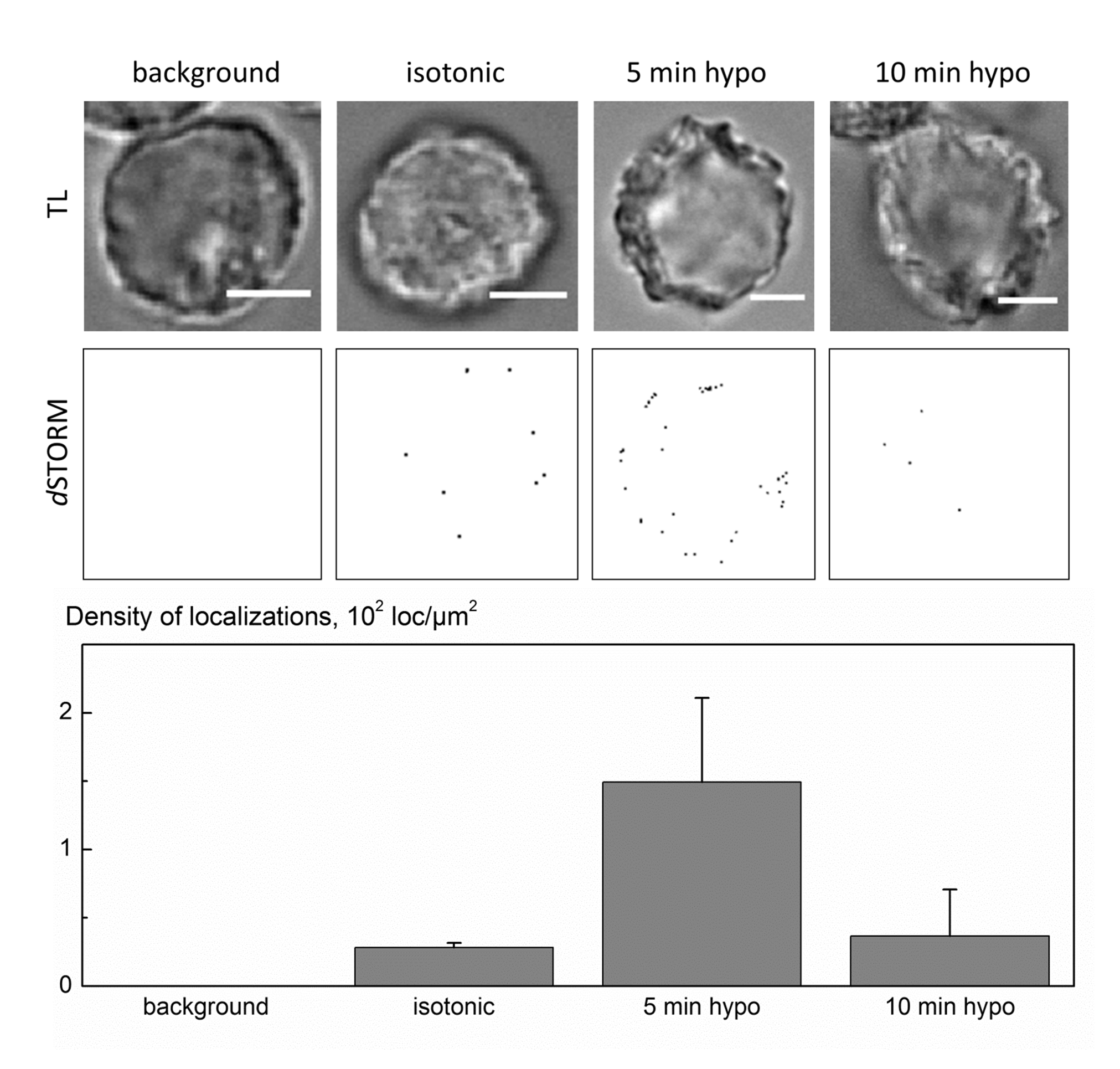

Supplement: S1 Fig — In these experiments, the cells were treated with a strongly hypotonic 100-mOsm sucrose solution for 5 and 10 min. In contrast to the experiments with 100-mOsm myo-inositol presented in Fig. 5, the cells underwent RVD in the presence of the disaccharide sucrose, as evident from Fig. 1B. (For further detail see Legend to Fig. 5) (TIF) [file pone.0119990.s001.tif]

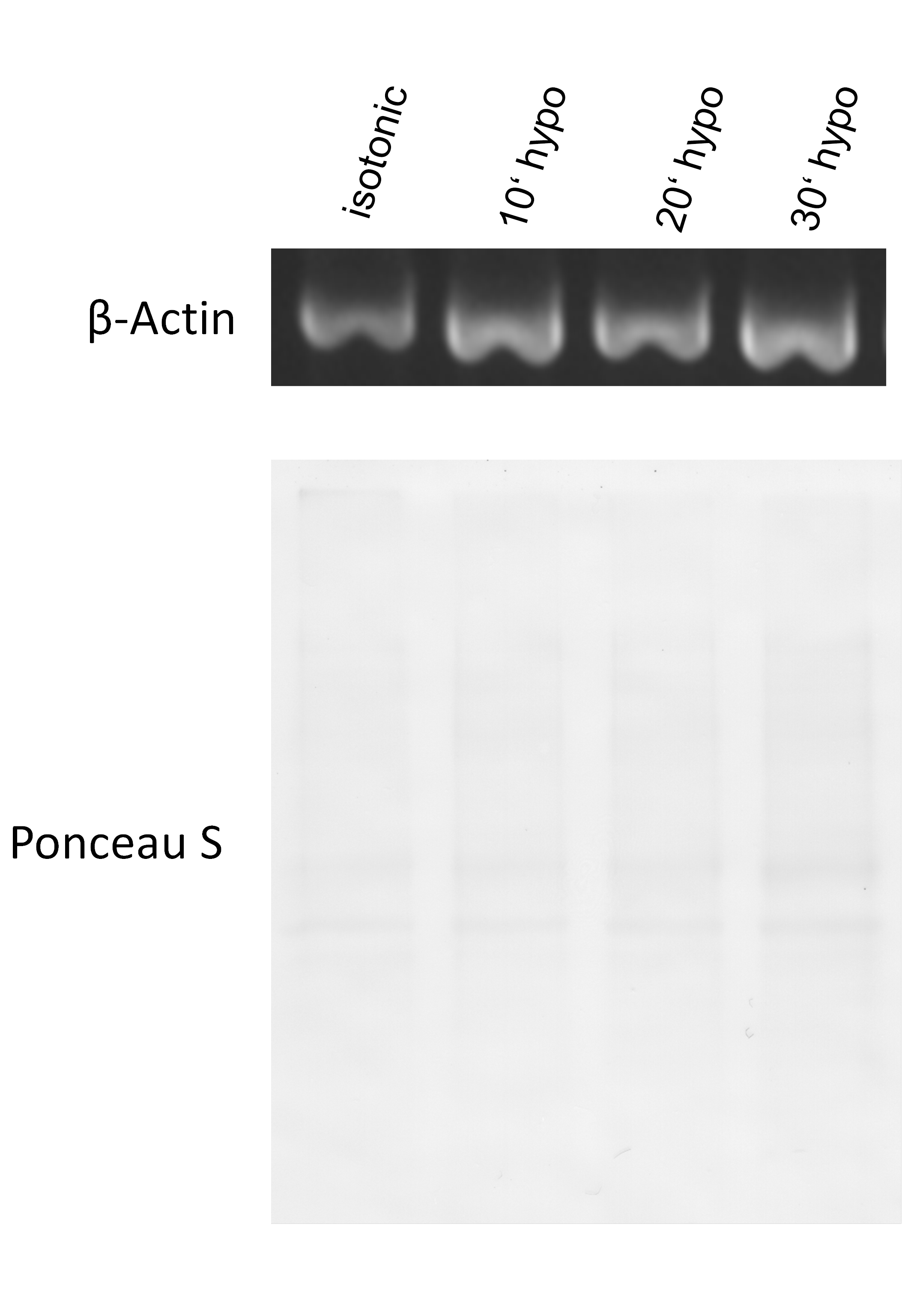

Supplement: S2 Fig — For further detail see text and Legend to Fig. 6. (TIF) [file pone.0119990.s002.tif]
