# Supplementary material for: Hypotonic Activation of the Myo-Inositol Transporter SLC5A3 in HEK293 Cells Probed by Cell Volumetry, Confocal and Super-Resolution Microscopy
Source: PLoS One. 2015 Mar 10;10(3):e0119990. doi: 10.1371/journal.pone.0119990 (PMC4355067; doi:10.1371/journal.pone.0119990)
Supplement: S1 Text — (DOCX) [file pone.0119990.s004.docx]

**Model of swelling-activated myo-inositol transport**

As evident from Fig. 1, *equiosmotic* replacement of sucrose by inositol at tonicities below 200 mOsm not only abolished RVD but also induced a considerable secondary cell swelling. Unlike the initial hypotonic swelling caused by an osmotic shift (e.g. 300 → 100 mOsm), the secondary swelling occurred under isosmotic conditions, i.e. *no* osmotic pressure gradient existed across the cell membrane. In our experiments, the isosmotic cell swelling implies a diffusion-driven influx of the major extracellular solute myo-inositol into cells. In contrast, the isosmotic cell shrinkage by RVD involves the release of intracellular electrolytes.

The following set of equations based on the two-parameter formalism [Kleinhans, 1998, Shirakashi et al., 2004] can be used for modeling isosmotic changes in cell volume:

$(C_{el}^{o}+C_{ ino}^{o})-(C_{el}^{i}+C_{ino}^{i})=0$ (A1),

$\frac{d\left(C_{ino}^{i}(\nu-\varepsilon)\right)}{dt}=P_{ino}(C_{ino}^{o}-C_{ino}^{i})\frac{S}{V_{0}}$ (A2),

$\frac{d\left(C_{el}^{i}(\nu-\varepsilon)\right)}{dt}=P_{el}(C_{el}^{o}-C_{el}^{i})\frac{S}{V_{0}}$ (A3),

where *C’s* are molar solute concentrations [mol/m^3^]; *V*_0_ and ν=V/V_0_ are the isotonic and normalized cell volumes, respectively; ε is the osmotically inactive volume fraction under isotonic conditions; *S* is the cell membrane area [m^2^] (assumed invariable); *P*’s are the membrane permeability coefficients [m/s]. The *subscripts* “*el”* and “*ino*” denote, respectively, *electrolyte* and *inositol*. The superscripts “*o*” and “*i*” stand for extra- and intracellular concentrations, respectively.

Equation A1 describes an osmotic equilibrium between the cytosol and external solution. Equation A1 implies that only two solutes, i.e. myo-inositol and electrolyte, maintain the osmotic equilibrium and that the membrane permeability to water is much higher than its permeability to either solute. Equation A2 relates the flux of myo-inositol across the cell membrane to the difference of its concentrations $\left( C_{ino}^{o}-C_{ino}^{i} \right)$ on the two sites of the membrane. Equation A3 is the corresponding relation for electrolytes. Combination of Eqs. A1-A3 leads to the following expression:

$\{(C_{ino}^{o}-C_{ino}^{i})+(C_{el}^{o}-C_{el}^{i})\}\frac{S}{V_{0}}=\frac{1}{P_{ino}}\frac{d\{C_{ino}^{i}(v-\varepsilon)\}}{dt}+\frac{1}{P_{el}}\frac{d\{C_{el}^{i}(v-\varepsilon)\}}{dt}=0$ (A4)

Given that $C_{el}^{i}=C_{el}^{o}+C_{ ino}^{o}-C_{ino}^{i}$ (Eq. A1), as well as *C*^o^_el_ and *C*^o^_ino_ are constant, Eq. A4 can be transformed to:

$(\frac{1}{P_{el}}-\frac{1}{P_{ino}})\frac{d\{C_{ino}^{i}(v-\varepsilon)\}}{dt}=\frac{1}{P_{el}}(C_{el}^{o}+C_{ino}^{o})\frac{d(v-\varepsilon)}{dt}$. (A5).

Combination of Eqs. A5 and A2 leads to the following expression:

$(\frac{1}{P_{el}}-\frac{1}{P_{ino}})P_{ino}(C_{ino}^{o}-C_{ino}^{i})\frac{S}{V_{0}}=\frac{1}{P_{el}}(C_{el}^{o}+C_{ino}^{o})\frac{d(v-\varepsilon)}{dt}$ (A6),

which in turn simplifies to

$(P_{ino}-P_{el})(C_{ino}^{o}-C_{ino}^{i})\frac{S}{V_{0}}=(C_{el}^{o}+C_{ino}^{o})\frac{d(v-\varepsilon)}{dt}=(C_{el}^{o}+C_{ino}^{o})\frac{dv}{dt}$ (A7)

Finally, the substitution of (*C^o^_ino_* - *C^i^_ino_*) with (*C^o^_el_* - *C^i^_el_*) (Eq. A1) transforms Eq. A7 to:

$(P_{el}-P_{ino})(C_{el}^{o}-C_{el}^{i})\frac{S}{V_{0}}=(C_{el}^{o}+C_{ino}^{o})\frac{dv}{dt}$ (A8),

In case of the membrane-impermeable sucrose, *C*^o^_ino_, *C*^i^_ino_ and *P*_ino_ can be replaced, respectively, by *C*^o^_suc_, *C*^i^_suc_=0 and *P*_suc_=0 in Eqs. A1 and A7, thus leading to:

$(C_{el}^{o}-C_{el}^{i})+C_{suc}^{o}=0$ (A9)

$-P_{el}C_{suc}^{o}\frac{S}{V_{0}}=(C_{el}^{o}+C_{suc}^{o})\frac{dv}{dt}$ (A10).

For spherical cells, the surface-to-volume ratio *S*/*V*_0_ = 3/*R*_0_, where *R*_0_ is the cell radius and Eq. A10 can be rewritten as:

$P_{el}=-\frac{R_{0}(C_{el}^{o}+C_{suc}^{o})}{3C_{suc}^{o}}\left(\frac{dv}{dt}\right)_{RVD}$ (A11).

Equation A11 (or Eq. 1 in the main text) was used to calculate the *P*_el_ during RVD. For these calculations we used the cell radius *R*_0_ = 7.9 µm; $C_{el}^{o}=15 mOsm$ and $C_{suc}^{o}=(85-285) mOsm$, according to experimental conditions. The rate of RVD Δν/Δ*t*_RVD_ was determined from the slope of volumetric curves, as illustrated in Fig. 1 (*blue lines*).

If during RVD, sucrose is replaced by an equiosmotic amount of myo-inositol, Eq. A10 is no longer valid. Instead, cell volume changes in the presence of membrane-permeable myo-inositol can be described by Eqs. A1, A7 and A8. The initial condition $C_{ino}^{i}=0$(for the time point just after the solution exchange) simplifies Eq. A8 to:

$(P_{ino}-P_{el})C_{ino}^{o}\frac{S}{V_{0}}=(C_{el}^{o}+C_{ino}^{o})\frac{dv}{dt}$ (A12).

Equation A12 can be rewritten as follows:

$P_{ino}=\frac{R_{0}}{3}\frac{(C_{el}^{o}+C_{ino}^{o})}{C_{ino}^{o}}\left(\frac{dv}{dt}\right)_{ino}+P_{el}$ (A13).

Eq. A12 and Eq. A13 (or Eq. 2 in the main text) are valid only for a short time after the replacement of sucrose by myo-inositol, where the initial condition $C_{ino}^{i}\approx0$is fulfilled.

In Eq. A13 (or Eq. 2 in the main text), the HEK293 cell radius *R*_0_ = 7.9 µm; $C_{el}^{o}=15 mOsm$ and $C_{ino}^{o}=(85-285) mOsm$, according to our experimental conditions. The rate of myo-inositol-mediated secondary swelling (Δν/Δ*t*_ino_) was determined from the volumetric data, as illustrated in Fig. 1 (*red lines*). The corresponding electrolyte permeability *P*_el_ was determined from the RVD rate (Eq. A11) at the time point of solute exchange (*blue fitted lines* in Fig. 1).
